# Protective Properties of Copper-Loaded Chitosan Nanoparticles against Soybean Pathogens *Pseudomonas savastanoi* pv. *glycinea* and *Curtobacterium flaccumfaciens* pv. *flaccumfaciens*

**DOI:** 10.3390/polym15051100

**Published:** 2023-02-22

**Authors:** Rashit Tarakanov, Balzhima Shagdarova, Tatiana Lyalina, Yuliya Zhuikova, Alla Il’ina, Fevzi Dzhalilov, Valery Varlamov

**Affiliations:** 1 Department of Plant Protection, Russian State Agrarian University—Moscow Timiryazev Agricultural Academy, 127434 Moscow, Russia; 2 Research Center of Biotechnology, Russian Academy of Sciences, 119071 Moscow, Russia

**Keywords:** copper-loaded chitosan nanoparticles, seed treatment, antibacterial properties, *Pseudomonas*, *Curtobacterium*, bacterial tan spot, bacterial blight

## Abstract

Soybeans are a valuable food product, containing 40% protein and a large percentage of unsaturated fatty acids ranging from 17 to 23%. *Pseudomonas savastanoi* pv. *glycinea* (Psg) and *Curtobacterium flaccumfaciens* pv. *flaccumfaciens* (Cff) are harmful bacterial pathogens of soybean. The bacterial resistance of soybean pathogens to existing pesticides and environmental concerns requires new approaches to control bacterial diseases. Chitosan is a biodegradable, biocompatible and low-toxicity biopolymer with antimicrobial activity that is promising for use in agriculture. In this work, a chitosan hydrolysate and its nanoparticles with copper were obtained and characterized. The antimicrobial activity of the samples against Psg and Cff was studied using the agar diffusion method, and the minimum inhibitory concentration (MIC) and minimum bactericidal concentration (MBC) were determined. The samples of chitosan and copper-loaded chitosan nanoparticles (Cu^2+^ChiNPs) significantly inhibited bacterial growth and were not phytotoxic at the concentrations of the MIC and MBC values. The protective properties of chitosan hydrolysate and copper-loaded chitosan nanoparticles against soybean bacterial diseases were tested on plants in an artificial infection. It was demonstrated that the Cu^2+^ChiNPs were the most effective against Psg and Cff. Treatment of pre-infected leaves and seeds demonstrated that the biological efficiencies of (Cu^2+^ChiNPs) were 71% and 51% for Psg and Cff, respectively. Copper-loaded chitosan nanoparticles are promising as an alternative treatment for bacterial blight and bacterial tan spot and wilt in soybean.

## 1. Introduction

Soybean (*Glycine max* (L.) Merr.) is a legume crop in the *Fabaceae* family. The importance of this crop stems from the fact that it is a valuable source of high-quality protein for human and livestock nutrition. Soybeans are a complete source of protein with essential amino acids as well as unsaturated fatty acids, dietary fiber, isoflavones, anthocyanins and vitamins [1]. In 2020, 353.5 million tons of soybean were harvested from 126.9 million ha worldwide, with an average yield of 27.8 q/ha [2]. However, yield growth is limited by several factors, most notably crop infestation, pests and diseases. More than 45 species of fungi, 15 species of viruses and 6 species of phytopathogenic bacteria cause economically significant diseases in soybean [3,4].

Bacterial infestation reduces yields by up to 40% and is the most destructive disease [5]. The Gram-negative bacterium *Pseudomonas savastanoi* pv. *glycinea* syn-*Pseudomonas syringae* pv. *glycinea* (Psg) is the causative agent of soybean bacterial blight [6]. At the present time, the area of distribution of the disease includes all the climatic zones and 41 countries in which the disease has been detected [7]. Psg not only causes specific symptoms on the upper leaves and pods, but it can also infect all above-ground parts of the soybean. Symptoms of the disease include the appearance of oily necrotic spots surrounded by chlorotic halos that gradually coalesce to form zones of necrosis [8]. Infected seeds and, more rarely, plant residues are the reservoirs of infection. This disease can reduce the germination of infected seeds and the yield and the content of unsaturated fatty acids [9].

Another disease affecting soybean is bacterial spot and wilt caused by the Gram-positive bacterium *Curtobacterium flaccumfaciens* pv. *flaccumfaciens* (Cff). The bacterium is able to cause spotting on leaves, burns and the death of seedlings and adult plants as well as penetrate into the vascular system [10]. The main symptoms of infection are slow growth, dying off of shoots, burns and wilting of stems. This pathogen can affect a wide range of leguminous crops, including soybean [11], although the main host plant is the common bean (*Phaseolus vulgaris* L.). Cff leads to a decrease in the yield and quality of seeds [12,13]. Cff is listed by the European and Mediterranean Plant Protection Organization (EPPO) as a quarantine object of category A2 [14]. The main source of infection is infected seeds [15].

The control of bacterial diseases in soybean requires a complex approach. The primary source of inoculum for bacterial diseases is infected plant residues. Infested seeds are a secondary source of inoculum; therefore, their certification is necessary in order to prevent their entry into the field [9,16]. Other methods for controlling bacterial diseases include strict crop rotation, the use of resistant varieties, and the treatment of plants and seeds with biological and chemical agents [17,18,19]. As decided by the European Union, the synthetic pesticides with the highest toxicity should be replaced by substances with a lower environmental impact in order to phase out their use (Implementing Regulation (EU) 2015/408) [20]. Therefore, the use of chitosan and chitosan-based compounds against bacterial diseases is a promising approach [21].

Chitosan is a biodegradable, biocompatible and low-toxicity biopolymer characterized by antimicrobial, antiviral, antioxidant, sorption and chelating properties [22,23,24,25]. Chitosan is a polycation in acidic pH. The biopolymer is obtained by chemical deacetylation of chitin under alkaline conditions. Chitin is one of the most common polysaccharides found in crustacean shells, insect cuticles and the cell walls of fungi [26]. The protective effect of chitosan is demonstrated by a triple action: activation of host defenses, effect on microorganisms and film formation on the treated surface [27]. The enhancement of plant immune response under the action of chitosan is due to the fact that positively charged chitosan can interact with negatively charged pectin. Plant cells receive information about the destruction of the cell wall and the presence of pathogens by inducing a specific alarm signal arising from chitosan’s effect on the supramolecular pectin structure. Plants react to chitosan–pectin dimeric complexes stronger than to individual components [28]. Chitosan can also directly inhibit the growth of many plant pathogens: phytopathogenic fungi [29], oomycetes [30] and bacteria [31]. Chitosan forms a protective film preventing the interaction of pathogens with the plant cell wall [32]. A number of commercial products based on chitosan, such as Armour-Zen (New Zealand), Chito Plant (Germany) and KaitoSol (United Kingdom) are used to inhibit the incidence of bacterial diseases in plants [33].

The application of chitosan nanoforms in the control of plant pathogens has become a trend in recent years [34]. Chitosan nanoparticles act as plant growth stimulators and antimicrobial agents against phytopathogenic microorganisms [35,36]. The mechanism of the antibacterial activity of chitosan nanoparticles is similar to that of chitosan and is primarily due to interaction with the cell wall and the bacterial cell membrane. In the case of chitosan nanoparticles, higher zeta potential values have a significant effect on bacterial growth inhibition when compared to the original forms of chitosan. In this vein, the smaller size and higher zeta potential of chitosan nanoparticles provide a higher level of antibacterial activity and attract increased interest from researchers as a means of combating bacteria [34,37]. Nanocomplexes of chitosan with metals, particularly with copper, are also actively studied. In the work [38] it was shown that copper-loaded chitosan-based nanoparticles with a size of 89 nm effectively inhibited the growth of *Xanthomonas axonopodis* pv. *punicae*, which causes the bacterial blight of pomegranate, at 1000 ppm and remained on par with standard streptocycline at 500 ppm.

Information on the antibacterial activity of copper-loaded chitosan nanoparticles and unmodified chitosan against Psg is scarce. Earlier, we described the antibacterial properties of chitosan hydrolysate against Psg in vitro at a concentration of 0.3% (*v/v*) [39]. In article [40], Cu-chitosan NPs were shown to have high antibacterial activity against Psg under in vitro conditions at concentrations of 400 ppm and 1000 ppm. There is no information on the efficacy of chitosan and chitosan nanoparticles with Cu^2+^ against Cff.

The aim of this study was to determine the antibacterial activity of chitosan hydrolysate and chitosan nanoparticles with copper against *Pseudomonas savastanoi* pv. *glycinea* and *Curtobacterium flaccumfaciens* pv. *flaccumfaciens* and determine their effectiveness in the treatment of soybean seeds and plants artificially infected with bacteria.

## 2. Materials and Methods

### 2.1. Preparation of Chitosan Hydrolysate

Crab shell chitosan with a molecular weight (MW) of 1040 kDa and a deacetylation degree (DD) of 85% was purchased from Bioprogress (Shchelkovo, Russia). Chitosan hydrolysate (ChiH) was prepared by chemical depolymerization of crab shell chitosan using nitric acid as described previously [41], with some modifications. Briefly, 1 g of chitosan was dispersed in 20 mL of 6.5% nitric acid, incubated for 7 h at 70 °C with stirring, cooled to room temperature, and kept without stirring for 16 h at 23 °C. Then, the pH was adjusted to 5.0–5.2 with 25% ammonium hydroxide, and the mixture was diluted with distilled water to a final volume of 180 mL. The pH of obtained ChiH with concentration 5 mg/mL was 5.2.

To determine the MW and polydispersity index, DD ChiH was preliminarily dialyzed against H_2_O. The MW of ChiH was determined via high-performance gel permeation chromatography in an S 2100 Sykam chromatograph (Sykam, Eresing, Germany) using a separation column (8 mm × 300 mm; PSS NOVEMA Max analytical 1000 A) and a pre-column (8.0 mm × 50 mm) [42]. Pullulans were used as calibration standards. The DD of Chi was determined using proton nuclear magnetic resonance (^1^H-NMR). Samples were prepared in deuterated water, and proton spectra were recorded on a Bruker AMX 400 spectrometer (Bruker, Watertown, MA, USA); 4,4-dimethyl-4-silapentane-sulfonic acid was used as a standard.

### 2.2. Preparation and Characterization of Chitosan Nanoparticles and Copper-Loaded Nanoparticles 

Chitosan nanoparticles (ChiNPs) were formed using the ionotropic gelation method as described previously [43] with some modifications. Chi (2 g) with MW = 39 kDa, DD = 90% and a polydispersity index of 2.4 was solved in 300 mL of 1% acetic acid. The chitosan solution was filtered through a glass filter to remove mechanical impurities. Tripolyphosphate (TPP) solution (Sigma-Aldrich, Munich, Germany) (5 mg/mL) was added dropwise under vigorous stirring until opalescence occurred (A = 0.100, λ = 590 nm), which was estimated using a Spekol 11 spectrophotometer (Carl Zeiss Jena, Germany). Copper-loaded chitosan nanoparticles (Cu^2+^ChiNPs) were obtained by dropwise addition of 25 mg/mL CuSO_4_ solution up to A = 0.144 (λ = 590 nm). NP preparations were adjusted with 1% acetic acid to a concentration of Chi = 5 mg/mL, CuSO_4_ = 0.83 mg/mL, pH 4.0. For the biological experiments, the ChiNPs and Cu^2+^ChiNP particles were not further purified and used as nanoparticles contained within suspensions. To characterize the particles, the suspension was preliminarily centrifuged for 10 min at 1000× *g* and then the supernatants were centrifuged at 14,000× *g* for 20 min to separate the NP fraction. The yield of ChiNPs was 8%, and that of Cu^2+^ChiNPs was 10%.

The mean hydrodynamic diameter of the NPs was determined via dynamic light scattering (DLS) in reflected light (scattering angle 180°) using a NANO-flex II analyzer (Colloid Metrix, Meerbusch, Germany), sample temperature 25 °C. The zeta potential of the NPs was characterized via DLS using a Zetasizer Nano (Malvern Panalytical, Malvern, UK); all measurements were performed at 25 °C, and the scattering angle was equal to 173°. NP suspensions were preliminarily centrifuged (Centrifuge 5418, Eppendorf, Hamburg, Germany) at 1000× *g* for 10 min, and then the supernatants were centrifuged at 14,000× *g* for 20 min at RT to separate the NP fraction.

Dimensional characteristics of the NPs were explored using an atomic force microscope, INTEGRA Prima (NT-MDT SI, Zelenograd, Russia). Scanning was performed with a resolution of 512 × 512 points in the semicontact mode in air. The scanning frequency was 1.3 Hz. Golden NSG01 silicon probes (TipsNano, Zelenograd, Russia) with a tip average resonance frequency of 150 kHz, an average force constant of about 5.1 N/m, and a cantilever curvature radius of 6 nm were used. The data obtained via AFM were visualized using software NOVA 1.0.26.860 (NT-MDT SI, Zelenograd, Russia), analyzed using Image Analysis 3.5.0.2069 (NT-MDT SI, Zelenograd, Russia) and processed with OriginPro B.9.2.196 (OriginLab Corporation, Northampton, MA, USA).

### 2.3. Bacterial Strains

The strains on which the experiments were carried out (strains Psg CFBP 2214 and Cff CFBP 3418) were obtained from the CFBP collection (Beaucouzé, France) and isolated and characterized by us in previous articles (Psg: G2 and G17, Cff: F-125-1 and F-30-1) [44,45]. These strains were pathogenic in soybean plants cv. Kasatka. The characterization of the Psg strains was performed by PCR analysis of the *cfl* (coronafacate ligase) gene [8] and analysis of the relationship of nucleotide sequences for the *cts* (citrate synthase) gene [46] with pathogen strains available in Genbank. Isolates belonging to Cff were determined via PCR analysis with genus-specific [47] and species-specific [48] primers.

### 2.4. Determination of Antibacterial Activity of Chitosan Samples

#### 2.4.1. Determination of Antibacterial Activity via Agar Diffusion Method

The agar diffusion method [49] was used for the primary determination of antibacterial activity using all six strains mentioned above. Briefly, 100 μL of bacterial suspension with a concentration of 1 × 10^8^ CFU/mL was applied to King’s B medium, distributed with a sterile loop, and wells 8 mm in diameter were pierced with a sterile cork borer. The bottoms of the wells were sealed by pouring a drop of molten King’s B medium (1.5% agar) into them. Then, 100 µL of sample was added to each at concentrations of 1, 5, 10, 25, 50, and 100% of the initial solutions. Samples with different concentrations were obtained by diluting the initial (100% solutions) in sterile water according to Table 1. The dishes were left at 4 °C for the diffusion of the solutions into agar (2 h) and then incubated at 28 °C for 48 h; ChiH and CuSO_4_ solutions were used as controls. The experiment was repeated three times.

#### 2.4.2. Determination of Minimum Inhibitory Concentration (MIC) 

MIC was determined according to [50] with modifications for all strains used in the article. In a 96-well sterile microtiter plate (Corning, Glendale, CA, USA), serial twofold dilutions of analyzed samples in King’s B liquid medium were prepared; the volume was 100 µL. After the dilutions, 70 µL of bacterial suspension at a concentration of 10^4^ CFU/mL was dissolved in King’s B liquid medium, 30 µL of 0.02% resazurin was added to each cell, and the mixture was thoroughly mixed. After 24 h, the plates were visually evaluated. The growth of bacteria in the medium was indicated by a color change from purple to pink. The lowest concentration at which a color change was observed was recorded as MIC. The experiment was repeated three times.

#### 2.4.3. Determination of Minimum Bactericidal Concentration (MBC)

The bactericidal activity of the chitosan samples in relation to all Psg and Cff strains used in the article was evaluated in accordance with the method of microdilution of broth described in CLSI 2015 [51] with modifications. For this, serial twofold dilutions of chitosan samples in King’s B liquid medium were prepared in a 96-well microtiter plate (Corning, Corning, NY, USA), and the volume was 100 µL. After the dilutions, 100 µL of bacterial suspension at a concentration of 10^4^ CFU/mL was added to each cell and the contents were thoroughly mixed. The plates were sealed with parafilm and incubated on a shaker-incubator, ES 20 (Biosan, Riga, Latvia), at 180 rpm and 28 °C. After 24 h of cultivation, 10 µL of bacterial suspension from each cell was tenfold diluted in sterile water and dispersed onto YD agarized medium (YDC without CaCO_3_) for subsequent titer calculation after 48 h. The experiment was repeated four times. A statistical analysis based on the results of the determination of MIC and MBC was not carried out because there were no differences within the repetitions.

#### 2.4.4. Determination of Time–Kill Curves

Time–kill curves were determined as described in [52] with some modifications. One colony of each bacterium (Psg CFBP 2214 and Cff CFBP 3418) was pre-cultured in 4 mL of King’s B liquid medium for 12 h at 28 °C and incubated on a shaker ES 20 (Biosan, Riga, Latvia) at 200 rpm. The cells were precipitated via centrifugation and titrated to a concentration of 10^4^ CFU/mL with sterile water. Bacterial titer control was carried out spectrophotometrically according to the OD_600_ index measured using a Nanodrop One (Thermo Fisher Scientific, Waltham, MA, USA). The cell suspension was then transferred to 1.5 mL sterile test tubes, and preparations were added to concentrations of 1xMBC. After that, the tubes were placed in a Thermomixer C (Eppendorf, Hamburg, Germany) and cultivated at 27 °C and 350 rpm. After 0, 2, 5 and 30 min and 1, 2 and 24 h, 10 µL of the mixture was taken, diluted in sterile SPS buffer, and dispersed on King’s B agarized medium. Colonies were counted after 48 h of cultivation at 28 °C. A suspension without antimicrobial agents was used as a negative control. The experiment was repeated three times.

### 2.5. Phytotoxicity on Soybean Seeds and Plants

The phytotoxicity of the chitosan samples on soybean seeds was assessed by a germination test using the standard “over paper” method described by the International Seed Testing Association [53]. Soybean (cv. Kasatka) seeds were soaked in aqueous solutions of the chitosan samples at various concentrations for 10 min and then completely dried on sterile filter paper at room temperature under sterile conditions. The samples of (1) water, (2) ChiH, (3) Cu^2+^ChiNPs, (4) Cu^2+^ChiH, (5) ChiNPs and (6) CuSO_4_ were diluted to concentrations of 1, 5, 10, 25, 50 and 100% (Table 1) of the stock solutions using sterile water.

Seeds soaked in sterile water were used as a negative control. Next, the seeds were incubated at a temperature of 25 °C with constant humidity. On the 8th day after treatment (DAT), germination was assessed; if a sprout with a well-developed root grew from a seed, it was considered to have germinated. The average percentage of seed germination was determined for all repetitions. The length of the roots was measured with a caliper after counting the germination rate and separating the cotyledons. The experiment consisted of 3 repetitions of 50 seeds in each group.

To test the phytotoxicity of the chitosan samples on plants, soybeans were grown to phase R1 (beginning bloom) in a turf–perlite mixture (Vieltorf, Velikiye Luki, Russia) in plastic pots for plant cultivation (volume 1 L, AgrofloraPack, Vologda, Russia).

Plants were kept in a greenhouse at 28/22 °C (14 h day/10 h night) under natural light and watered as needed. The foliar treatment was carried out with tested samples using a sprayer (with a drop size of ~300 µm) at a consumption rate of a sample solution of ~5 mL/plant (until all leaves were completely wetted).

Phytotoxicity was assessed after 7 days of incubation under the same conditions, according to the phytotoxicity scale [54], where: 0—no symptoms; 1—very slight discoloration; 2—more severe, but short; 3—moderate and longer; 4—medium and long; 5—moderately severe; 6—heavy; 7—very heavy; 8—almost destroyed; 9—destroyed; 10—completely destroyed. The experiment was repeated three times, with two plants in each repetition. The phytotoxicity rating was considered as the average score for each variant (the sum of the scores of each leaf/the number of analyzed leaves).

### 2.6. Control Psg and Cff Artificial Infection by Chitosan Samples 

All experiments on the use of the chitosan samples in the artificial infection of soybean seeds and leaves with bacterial diseases were carried out from May to August 2022 under the conditions of an experimental greenhouse using the Kasatka soybean cultivar (harvest year 2021; weight of 1000 seeds = 122.8 g). In these experiments, the strains Psg CFBP 2214 and Cff CFBP 3418 were used.

#### 2.6.1. Control Psg on Seeds

Artificial Psg infection of seeds was carried out according to the method in [45]. Briefly, a 72 h culture of Psg CFBP 2214 was suspended in sterile 10 mM MgCl_2_ at ~10^4^ CFU/mL. Soybean seeds were sterilized in 75% ethanol for 2 min, washed with an aqueous 50% solution of commercial bleach (sodium hypochlorite)/0.002% Tween 20 (*v/v*) for 8–10 min and distilled H_2_O until the chlorine was removed, and left in a humid chamber for 2 h to make them swell. The swollen seeds were pierced with a sterile toothpick, transferred to a flask with a bacterial suspension, vacuum treated at −10^5^ Pa for 10 min and dried to remove excess liquid.

The infected seeds were immersed for 10 min in 50% solutions of (1) water, (2) ChiH, (3) Cu^2+^ChiNPs, (4) Cu^2+^ChiH, (5) ChiNPs and (6) CuSO_4_ (Table 1). After that, the seeds were dried on paper towels to get rid of excess moisture.

The treated seeds of the experiment were sown in a peat–perlite mixture (Veltorf, Velikie Luki, Russia) in 40-cell plastic seed trays (cell volume 0.12 L, AgrofloraPak, Vologda, Russia). The plants were watered as needed and grown in a greenhouse in natural sunlight at 28/22 °C (14 h day/10 h night). Treatments in each experiment were organized according to the scheme of complete randomization. Each treatment had 5 replications with 40 seeds (1 tray per replication).

#### 2.6.2. Control Psg on Leaves

Psg infection of soybean plants was carried out according to the method in [55], using suspension infiltration with a 1113 AirControl airbrush (JAS, Ningbo, China). The bacterial suspension was prepared in the same way as it was for seed inoculation, but with the addition of Silwet Gold surfactant (Chemtura, Philadelphia, PA, USA) at a concentration of 0.01% (*w/w*). Infection was carried out with an average dose of 5 mL of suspension with a concentration of 10^9^ CFU/mL per trifoliate leaf. Plants were cultivated according to Section 2.6.1. in 0.5 L pots. Each treatment had three replications with 10 plants per replication.

The design of the experiment included the use of (1) water, (2) ChiH, (3) Cu^2+^ChiNPs, (4) Cu^2+^ChiH, (5) ChiNPs (6) and CuSO_4_ (Table 1).

The percentage of plants that exhibited leaf symptoms was recorded. The LeafDoctor app (https://www.quantitative-plant.org/software/leaf-doctor, accessed on 21 July 2022) installed on an iPhone SE 2 was used to assess the development of the disease by the degree of infection of adult plants. For this, all plants were photographed and analyzed by moving the threshold slider until only symptomatic tissues were converted to blue and the percentage of affected tissue was calculated according to the developer’s recommendations [56]. The same calculations were made in the seed treatment experiment after reaching stage V3 (35 days after sowing).

#### 2.6.3. Control Cff on Seeds

Inoculation through hilum injury described in [16] with modifications was used for seed infection by Cff. For this purpose, the hilum of each seed was pierced with a sterile needle, soaked in a bacterial suspension, placed in a vacuum, and then dried on paper towels under sterile conditions.

Soybean seeds were treated via immersion for 10 min in an aqueous solution of (1) water, (2) ChiH, (3) Cu^2+^ChiNPs, (4) Cu^2+^ChiH, (5) ChiNPs and (6) CuSO_4_, then dried on paper napkins to get rid of excess moisture. Further actions with plants and growing conditions were similar to Section 2.6.1.

Bacterial wilt was scored for each plant at 15, 18, 21, 24, 27, and 31 days post-seeding on a scale of 0 to 5, where 0 = no wilt symptoms; 1 = wilting of one of the primary leaves; 2 = wilting of both primary leaves but not the first trifoliate; 3 = withering of the first trifoliate leaf; 4 = death of the seedling after the development of primary leaves; and 5 = no germination or complete wilting and loss of turgor (in adult plants) of soybean scales adapted by us in a previous study described in [57]. Using this scale and methodology [58], the AUPDC (area under progress disease curve) was calculated using MS Excel 2007.

#### 2.6.4. Control Cff on Leaves

The Cff infection of soybean plants and the method for calculating plant disease were similar to Section 2.6.2. The design of the experiment included the use of: (1) water, (2) ChiH, (3) Cu^2+^ChiNPs, (4) Cu^2+^ChiH, (5) ChiNPs and (6) CuSO_4_. The calculation of the incidence rate, replicate and plant growth conditions was similar to Section 2.6.2.

### 2.7. Statistical Analysis

For all experiments, data analysis was carried out using the analysis of variance method using Statistica 12.0 (StatSoft, TIBCO, Palo Alto, CA, USA), comparing the average values using Duncan’s criterion. The percentage data were converted to arcsine before processing. Graphs were created using GraphPad Prism 9.2.0 (GraphPad Software Inc., Boston, MA, USA).

## 3. Results and Discussion

### 3.1. Preparation Samples Based on Chitosan

Chitosan hydrolysate with the main fraction (MW 39 kDa, DD 90%, polydispersity index 2.4) was prepared from high-molecular-weight chitosan (MW 1040 kDa, DD 85%) by acid hydrolysis using nitric acid. We assume that the chitosan hydrolysate preparation considered in this work can be applied in practice in agriculture. In this regard, we attempted to simplify the method of preparation by not isolating a separate fraction of low-molecular-weight chitosan.

Along with the properties typical for Chi, ChiNPs had the valuable advantages of nanoparticles, namely their large surface area and small size [59,60]. Muthukrishnan et al. described the ability of chitosan nanoparticles to inhibit the growth of *Pyricularia grisea, Alternaria solani* and *Fusarium oxysporum* [61]. In the same work, chickpea seed treatment had positive morphological effects, such as an increase in germination percentage, seed strength index and vegetative biomass of seedlings.

The versatility of ChiNP activity against plant pathogens, particularly tomato, of both fungal and bacterial etiology was presented in [62]. It was shown that chitosan nanoparticles possessed antimicrobial activity towards a complex of tomato pathogens, which include fungi *Colletotrichum gelosporidies*, *F. oxysporum*, *Gibberella fujikuori, Sclerotinia sclerotiorum* and *Phytophthora capsici* and bacterium *Pectobacterium carotovorum* subsp. *carotovorum X. campestris* pv. *vesicatoria*. Recently, chitosan–metal nanocomplexes with improved antimicrobial activity were synthesized (Ag^+^-ChiNPs, Cu^2+^-ChiNPs, Zn^2+^-ChiNPs, Mn^2+^-ChiNPs and Fe^2+^-ChiNPs) [63,64,65,66]. The work [67] shows the activity of chitosan nanoparticles against a number of pathogens of bacterial plant diseases, including *Agrobacterium tumefaciens*, *Erwinia* sp. and *X. campestris* with MIC values of 100, 500 and 500 ppm, respectively. All these results indicate that chitosan nanoparticles can be used in the field to protect various crops from pathogens of different etiologies.

To obtain chitosan nanoparticles, the main fraction of hydrolysis (MW 39 kDa) was dialyzed and freeze-dried. ChiNPs were obtained by ionotropic gelation under acidic conditions (pH 4.0). The formation of ChiNPs occurred due to the interaction of positively charged chitosan amino groups with TPP, which has phosphate groups with a negative charge. In contrast to the original technique [43], low-molecular-weight chitosan was used; thus, it was possible to use a more concentrated solution of Chi, but the formed particles were larger. In our work, the ChiNPs and ChiH samples had the same chitosan concentration of 5 mg/mL.

To obtain Cu^2+^ChiNPs, copper sulfate was added to a suspension of nanoparticles up to a final concentration of 0.83 mg/mL. The formation of complexes can occur through adsorption, ion exchange and chelation. The interaction type is defined by the solution formulation, the pH value and the type of metal ion [68]. Chitosan is able to form complexes with some metal ions, predominantly through interactions with amino groups and hydroxy groups (especially in the C3 position), that promote sorption [69]. The dimensional characteristics and charge of the nanoparticles measured using the DLS method are shown in Table 2. The measurements of nanoparticle size were carried out using the particle number distribution. The hydrodynamic diameter of ChiNPs was larger compared to Cu^2+^ChiNPs. The polydispersity of nanoparticles purified from unbound polymer (ChiNPs cf) was lower than that of ChiNPs and Cu^2+^ChiNPs.

**Figure 1:**
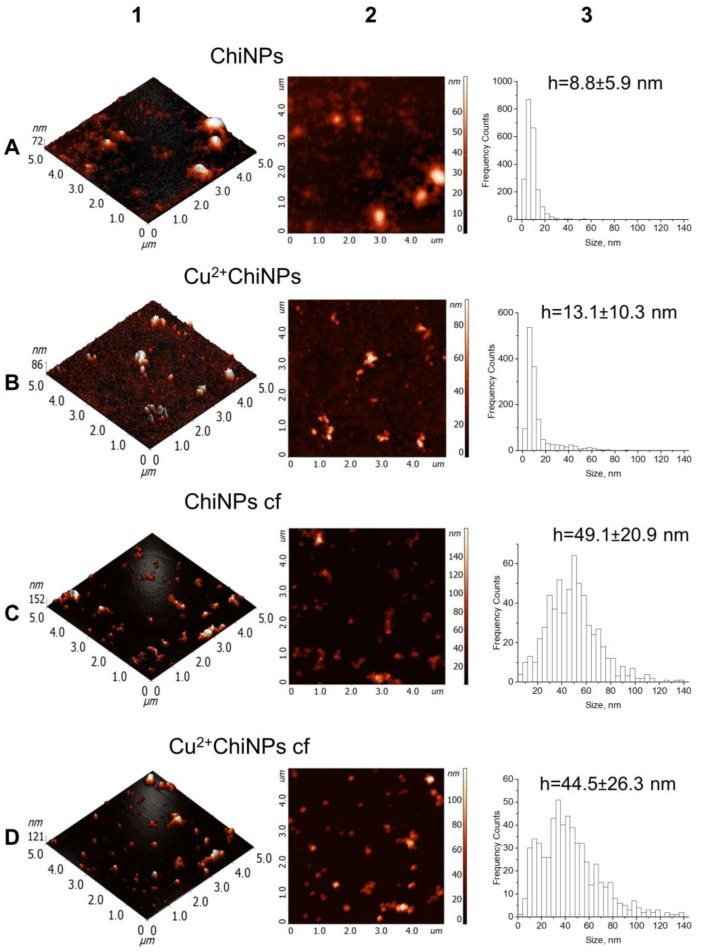
Dimensional characteristic of NPs. Column 1 (**A1**–**D1**)—3D AFM images of nanoparticles, column 2 (**A2**–**D2**)—2D AFM images of nanoparticles, column 3 (**A3**–**D3**)—histograms of the size distribution of nanoparticles and their average sizes according to AFM. Row **A**—ChiNPs, row **B**—Cu^2+^ChiNPs, row **C**—ChiNPs cf, row **D**—Cu^2+^ChiNPs cf.

The AFM method was used to characterize the size and morphology of the nanoparticles (Figure 1). The AFM-images of ChiNPs and Cu^2+^ChiNPs (Figure 1(A1,A2,B1,B2)) show that the suspension contained a large amount of unbound polymer forming aggregates smaller than 15 nm. To characterize the main fraction of nanoparticles, it was separated from the unbound polymer by centrifugation (ChiNPs or Cu^2+^ChiNPs were preliminarily centrifuged for 10 min at 1000× *g* and then supernatants were centrifuged at 14,000× *g* for 20 min to separate the NP fraction). As a result, the fractions of nanoparticles ChiNPs cf and Cu^2+^ ChiNPs cf were isolated (Figure 1(C1,C2,D1,D2)). There were no significant differences in the particle sizes of ChiNPs cf and Cu^2+^ChiNPs cf, which were 30–60 nm (Figure 1(C3,D3)). ChiNPs cf had an amorphous structure in contrast to the more compact structure of Cu^2+^ChiNPs cf. When comparing the morphology of the synthesized nanoparticles, it was found that ChiNPs cf had a greater tendency to aggregate.

We assumed that when scaling up the nanoparticle formation technology for agricultural use, it would not be advisable to isolate the nanoparticles fraction from the reaction mixture. Therefore, biological efficacy tests were carried out with crude nanoparticle preparations, which were a mixture of nanoparticles and an unbound polymer/Cu^2+^, but for brevity, we continued to use the abbreviations ChiNPs or Cu^2+^ChiNPs for nanoparticle-containing samples.

### 3.2. Antibacterial In Vitro Activity

The primary antibacterial activity of chitosan samples was tested using the agar diffusion method towards three *P. savastanoi* pv. *glycinea* strains and three *C. flaccumfaciens* pv. *flaccumfaciens* strains.

#### 3.2.1. Determination of Antibacterial In Vitro Activity via Agar Diffusion Method

**Figure 2:**
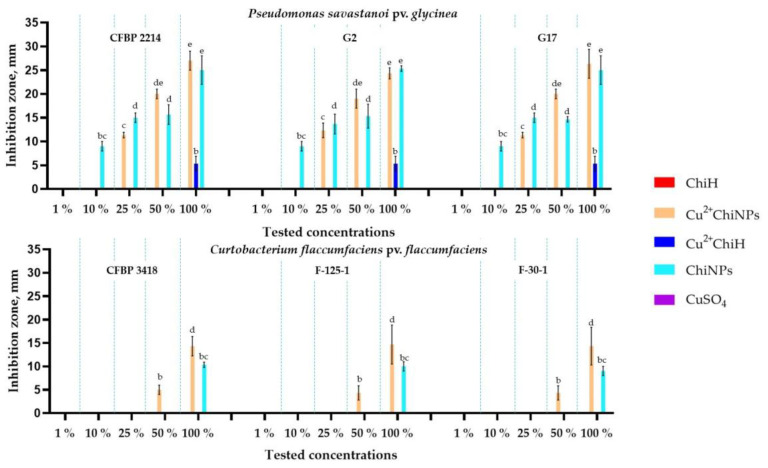
The diameters of the inhibition zones for each test substance, depending on the concentration of solutions against Psg and Cff strains (the average value for every strain of each bacterium) in an agar diffusion test. We added 100 µL of the sample to the well, and after 48 h of incubation at 28 °C, the zone of inhibition was measured. Different letters indicate a significant difference in values, according to Duncan’s test, at *p* = 0.05. All tests were carried out three times. The standard deviation (SD) is shown for each bar.

Pathogens had different sensitivities to chitosan that depended on the strain, sample type and dose (Figure 2). It should be noted that the analyzed substances exhibited a stronger antibacterial effect against Psg strains, whereas Cff strains were more resistant. Cu^2+^ChiNPs were the most effective at all analyzed concentrations; the diameter of the inhibition zone of 100% Cu^2+^ChiNPs suspension (5 mg/mL of chitosan and 0.83 mg/mL of copper) was 27 mm for Psg and about 15 mm for Cff (Figure S1). Although ChiH and CuSO_4_ exhibited no antibacterial activity (Figure 2), the average diameter of the inhibition zone for Cu^2+^ChiH (the combination of ChiH and CuSO_4_) on Psg strains was about 5 mm. The low efficiency of the CuSO_4_ solution can be explained by the low concentration. We suggest that the low effect of ChiH is due to the difficulty of diffusion of the chitosan polymer molecules in the nutrient medium at neutral pH, similar to the data reported in article [70]. It is most likely that the addition of copper sulfate to ChiH resulted in the formation of more compact complexes of chitosan with copper, which increased diffusion into the agar. For Cu^2+^ChiH, ChiNPs, and Cu^2+^ChiNPs, there were dose-dependent dynamics of increasing the zone of bacterial growth inhibition. Chitosan-based copper nanoparticles, obtained using a chemical reduction method, effectively inhibited growth of *X. axonopodis* pv. *punicae* at a concentration of 1000 [38], which is in agreement with our data.

#### 3.2.2. Determination of Minimum Inhibitory and Bactericidal Concentrations

The MIC and the MBC of the chitosan samples are shown in Table 3. It was found that the inclusion of copper in the nanoparticles led to a decrease in MIC and MBC in relation both to copper sulfate and ChiNPs. The addition of copper ions to chitosan hydrolysate (Cu^2+^ChiH) enhanced its antibacterial activity. However, the activity of Cu^2+^ChiH was lower compared to Cu^2+^ChiNPs.

Unfortunately, there are few works devoted to the study of the efficacy of chitosan nanoparticles loaded with copper against phytopathogenic bacteria. Therefore, we will also consider those works in which antibacterial activity was studied on human opportunistic bacteria. Du et al. investigated chitosan-based nanoparticles loaded with Cu^2+^ ions obtained via ionotropic gelation. On the bacteria *E. coli*, *S. choleraesuis* and *S. aureus*, it was shown that the antibacterial activity of such nanoparticles was significantly higher compared to the activity of chitosan nanoparticles and Cu^2+^ ions. In addition, Gram-negative bacteria were more sensitive than Gram-positive bacteria [71]. Antibacterial activity of CuO, Cu_2_O and Cu^0^ nanoparticles obtained by using reducing agents has also been studied. For CuO nanoparticles, the bactericidal concentration against *Ralstonia solanacearum* causing bacterial wilt was 250 µg/mL [72]. The MBC values for CuO nanoparticles were 100 µg/mL for *S. aureus* (MRSA), 250 µg/mL for *E. coli* and 5000 µg/mL for *P. aeruginosa* in [73]. These data are consistent with our data.

It was found that ChiH was less active compared to ChiNPs. This was probably due to the fact that in King’s B medium with a pH of 7.0–7.2, used in this test, the protonation of amino groups responsible for the manifestation of antibacterial activity decreases [35]. One of the mechanisms of chitosan action is considered to be its ability to form films around bacterial cells [74]. However, in our work, ChiH contained the main fraction with a low molecular weight, which decreases film-forming ability. Chitosan NPs exhibited higher antibacterial activity than chitosan, probably due to their higher surface-to-volume ratio and surface energy [35]. The higher activity of chitosan NPs compared to chitosan was previously reported by Qi et al. in [43].

From the CuSO_4_ and Cu^2+^ChiH test results, it is evident that the CFBP 2214 and G17 (Psg) strains had a greater sensitivity to copper than strain G2. This fact may be an indirect indicator of the diversity of strains, including sensitivity to bactericides in the country. MIC ChiH data show that bacteria of the Cff species were more sensitive to chitosan (78 mg/mL) compared to Psg (156 mg/mL). One of the possible reasons for these differences is the different structure of the bacterial cell wall. For example, in the paper [75], using four Gram-negative bacteria (*Escherichia coli*, *P. fluorescens*, *Salmonella typhimurium*, and *Vibrio parahaemolyticus*) and seven Gram-positive bacteria (*Listeria monocytogenes*, *Bacillus megaterium*, *B. cereus*, *Staphylococcus aureus*, *Lactobacillus plantarum*, *L. brevis*, and *L. bulgaricus*), it was shown that Gram-positive bacteria are more sensitive to chitosan.

#### 3.2.3. Antibacterial In Vitro Activity by Determination of Time–Kill Curves

**Figure 3:**
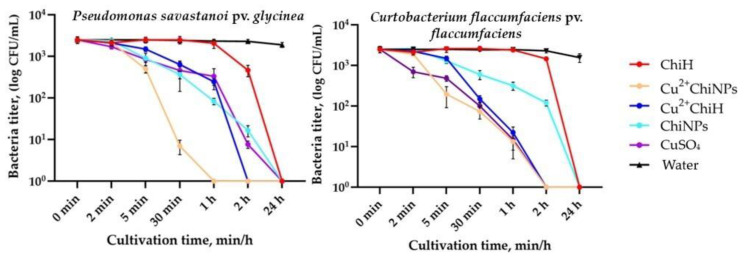
Time–kill curves for chitosan samples and CuSO_4_ for Psg CFBP 2214 and Cff CFBP 3418. A concentration of 1xMBC was used in all analyses. Error bars represent standard deviations (SDs) of the mean of the viable cells number (CFU/mL) for 3 independent repeats.

Another important parameter that determines the effectiveness of antibacterial agents is the rate of cell death, as described by time–kill curves. Figure 3 shows the time–kill curves for the Psg CFBP 2214 and Cff CFBP 3418 strains.

The experimental design was to determine the exposure time at which complete loss of cell viability occurred. In the case of Psg, Cu^2+^ChiNPs caused complete cell death within the first hour of cultivation. Cu^2+^ChiH acted within 2 h; for the other samples, 100% death was achieved after 24 h of exposure. The effect of all samples on Cff strains was achieved after 2 h, except for ChiNPs and ChiH, which caused 100% death in 24 h. Cell viability was virtually unchanged in the presence of water. A similar kinetic of ChiNPs action was shown by Dash et al., where complete killing of *B. subtilis* and *S. aureus* was not achieved within 4 h [76]. At the same time, in nearly all variants, 50% cell death occurred within 30 min. Thus, Cu^2+^ChiNPs exhibited the most rapid bactericidal effect, causing the complete death of bacteria in liquid nutrient medium within 1 h for Psg and 2 h for Cff. Christena et al. also found that CuNPs had a bactericidal effect on *S. aureus* at a concentration of 2xMIC and *P. aeruginosa* at a concentration of 1xMIC. Four hours after treatment with CuNPs, a five-fold logarithmic decrease in CFU was observed for *Staphylococcus,* and a three-fold logarithmic decrease in CFU was demonstrated for *Pseudomonas* [77]. Thus, the determination of time–kill curves shows that Cu^2+^ChiNPs have a greater potential to fight bacteria due to their high kill rate compared to the initial forms of chitosan and copper.

### 3.3. Phytotoxicity on Seeds and Leaves

To determine the limiting concentration of the samples for the treatment of soybean plants, phytotoxicity tests were performed at concentrations of 0, 25, 50, 75 and 100 % of the stock solutions (according to Table 1).

**Figure 4:**
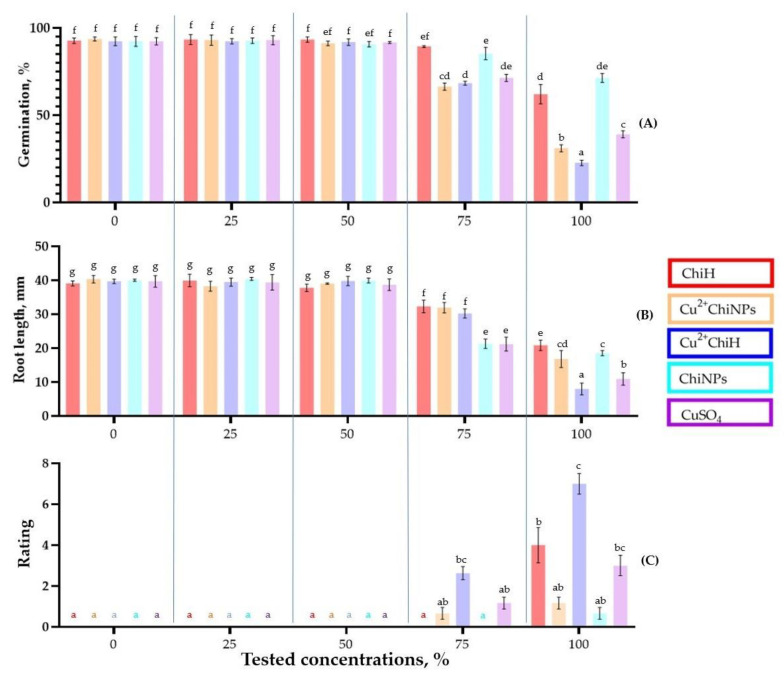
Phytotoxicity of chitosan samples on soybean leaves and seeds. Germination values (**A**) and root length (**B**) of soybean seeds after treatment with different concentrations of samples 8 d after treatment. The average score of the phytotoxicity integral value on soybean leaves for chitosan samples was measured at 72 h after treatment (**C**). Values represent the mean of three independent trials, error bars represent the standard deviation. Values marked by different letters have a significant difference, according to Duncan’s criteria, at *p* = 0.05.

The effect of the sample concentrations on seed germination and root length of soybean seedlings is shown in Figure 4A,B. The phytotoxicity of samples at various concentrations was determined by the average values of germination and root length. The obtained values were compared with a water-treated control. For all samples, the phytotoxic effect was observed at concentrations above 50% of the stock solutions, corresponding to 2.5 mg/mL of chitosan and 0.42 mg/mL of CuSO_4_.

At 50% concentration of the samples (2.5 mg/mL of chitosan and 0.42 mg/mL of CuSO_4_), an insignificant decrease in germination and root length was observed. When treated at initial concentrations (5 mg/mL of chitosan and 0.83 mg/mL of CuSO_4_), Cu^2+^ChiH had the strongest reduction in seed germination, and ChiNPs had the least phytotoxic effect. Cu^2+^ChiH had the most toxic effect on root length, and ChiH was the least toxic. It is important that the inclusion of copper in the nanoparticles increased their antibacterial activity and reduced the phytotoxicity of copper.

Phytotoxicity on soybean leaves was tested by spraying samples at different concentrations. For all samples, a dose-dependent increase in phytotoxicity with increasing concentration was determined.

As in the case of seeds, safe non-phytotoxic concentrations for leaf treatment were 50% of the initial solutions (2.5 mg/mL of chitosan and 0.42 mg/mL of CuSO_4_) for all analyzed samples (Figure 4C). Cu^2+^ChiH had the highest phytotoxicity; when treating with a 100% solution (5 mg/mL of chitosan, 0.83 mg/mL of CuSO_4_), phytotoxicity symptoms in the form of leaf blights were observed, with the average phytotoxicity score reaching 7.0, which corresponds to very heavy leaf damage (Figure 4C and Figure S2).

The high phytotoxicity of ChiH was probably due to the presence of salts in the form of ammonium nitrate and $NO_{3}^{-}$ as counter ions on the amino groups of chitosan. The phytotoxicity of copper in Cu^2+^ChiNPs was much lower compared to Cu^2+^ChiH and CuSO_4_ solution. This is probably due to the slow release of copper from the nanoparticles compared to CuSO_4_ solution, as confirmed in the study by Young et al. [78]. Sathiyabama et al. found no symptoms of phytotoxicity when finger millet (*Eleusine Coracana* (L.) Gaertn) was treated with copper–chitosan nanoparticle solution [79], which is consistent with our data. At the same time, metal particles without chitosan exhibited phytotoxic properties, such as in the work of Stampoulis et al., where treatment with copper nanoparticles (Cu^0^) at a concentration of 1 mg/mL resulted in a 90% reduction in biomass of zucchini plants compared to untreated control plants [80]. In contrast, Shende et al. found that treatment of pigeon pea (*Cajanus cajan* L.) with CuNP solution at a concentration of 20 ppm resulted in an increase in height, root length, fresh and dry weight and plant productivity index [81]. This may be due to both the lower copper concentration and the green method of particle production using plant extracts.

Thus, to comply with the principle of a single difference, further studies on the control of soybean bacterial diseases using chitosan-containing samples were performed using 50% solutions (2.5 mg/mL of chitosan and 0.42 mg/mL of CuSO_4_) that found no statistically significant indicators of phytotoxicity on soybean.

### 3.4. The Efficiency of Chitosan Samples against Psg and Cff Infection on Leaves and Seeds 

The repeatability of the «Psg-soybean» and «Cff-soybean» pathosystem models has been described and explained in detail in our previous publications [45,57], and the experimental conditions were identical. Soybean leaves preliminarily infected with Psg and Cff suspensions were treated with chitosan samples. Disease spread on soybean leaves was measured 12 days after treatment using Leaf Doctor software.

**Figure 5:**
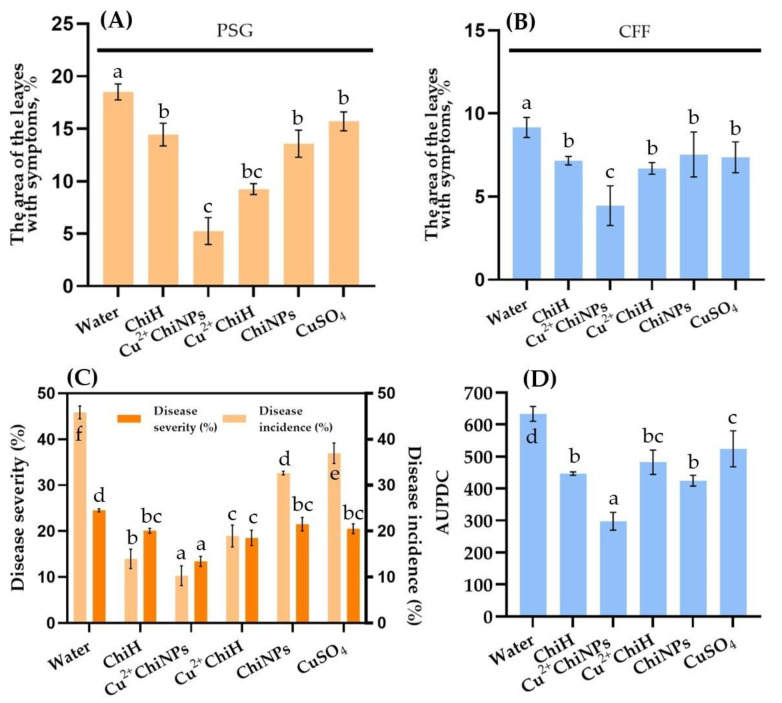
Bacterial blight (**A**,**C**) and bacterial tan spot and wilting (**B**,**D**) of soybean, caused by artificial inoculation of Psg and Cff after treatment with chitosan samples. (**A**,**B**): disease severity on inoculated green plants; (**C**): disease severity and incidence after inoculation of soybean seed by Psg; (**D**): values of AUPDC after inoculation of soybean seeds by Cff. Values are averages from three independent tests, error bars show standard deviation. Columns with a significant difference are marked with different letters, Duncan’s test, *p* = 0.05.

Chitosan samples reduced the degree of leaf lesions from Psg by 15–71% compared with water-treated controls (Figure 5A,B). Cu^2+^ChiNPs resulted in a 71% decrease in lesion area compared to controls, while Cu^2+^ChiH contributed only up to 50%. Treatment with CuSO_4_, ChiH and ChiNPs did not cause a significant reduction in leaf lesions (15–20%) compared to control.

The average leaf area with disease symptoms in the control group infected with Cff was inferior to the Psg infected group but remained at a high level (9.2% and 18.5%, respectively). The highest efficiency was observed for Cu^2+^ChiNPs (51.3% reduction of lesion area), while the efficiency of other samples ranged from 17.8 to 26.9% compared with control (Figure S3).

Treatment of soybean seeds pre-infected with Psg using the chitosan samples exhibited a significant decrease in seedling infection frequency and disease development rate.

In the case of water-treated plants, rapid disease development was observed (Figure 5C). With daily overwatering of plants, a secondary infection was created, similar in severity to an outbreak of the disease in the field.

The biological effectiveness of Cu^2+^ChiNPs treatment was 77% (disease incidence) or 45.3% (disease severity) compared to control. Cu^2+^ChiH treatment reduced disease development ~1.3-fold and disease incidence more than 2-fold. CuSO_4_ solution and ChiNPs treatments were the least effective. Their effectiveness on disease development was 19.3% and on disease incidence 16.3%. The Cff infected control group of seeds exhibited symptoms of wilting and yellowing of soybean leaves with an average AUPDC = 609 score (Figure 5D). In general, the treatment efficacy of all samples was lower for Cff than for Psg. Thus, the Cu^2+^ChiNPs treatment was the best, with a biological efficiency of 53% compared to the control, while ChiNPs reduced AUPDC by only 33%. Treatment of seeds with CuSO_4_ demonstrated a low biological effect; the efficiency was 17%.

The effectiveness of treatment of plants with chitosan copper-loaded nanoparticles strongly depends on many factors, one of which is the concentration of active substances and the type of pathogen. For example, in the work of Swati et al., the treatment of soybean plants with Cu-chitosan NP at a concentration of 0.02–0.12% reduced the severity of the bacterial pustule by 50.0–33.3% and 55.3–34.0% in the pot and in the field, respectively [82]. Kumar et al. studied the effectiveness of copper–chitosan-based nanoparticles in the treatment of banana plants against *F. oxysporum* f. sp. *cubense*. At a concentration of 0.20 mg/mL, high efficacy was shown, which amounted to a 73% reduction in symptoms compared to the untreated control [83].

Thus, our results demonstrate the protective effects of copper-loaded chitosan nanoparticles on soybean seed and leaf from bacterial blight and rust-brown bacterial spot and wilt. Further research is needed to improve the efficacy of soybean treatments by optimizing delivery technology, determining biosafety and developing the formulation for commercial use.

## 4. Conclusions

In this article, the synthesis of different chitosan samples (chitosan hydrolysate, chitosan hydrolysate with copper, chitosan nanoparticles and copper-loaded chitosan nanoparticles) and evaluation of their antibacterial action in vitro and in an artificial infection of soybean bacterial diseases were carried out.

The Cu^2+^ChiNPs sample demonstrated the greatest antibacterial activity, with maximum inhibition zone diameters of 27 mm and 15 mm and the shortest total bacterial kill times of 1 h and 2 h for *Pseudomonas savastanoi* pv. *glycinea* and *Curtobacterium flaccumfaciens* pv. *flaccumfaciens*, respectively. Evaluation of all samples for their phytotoxicity by treatment of soybean leaves and seeds demonstrated that they are safe for soybean plants at the concentrations of 2.5 mg/mL of chitosan and 0.42 mg/mL of CuSO_4_ or less.

In the process of studying the protective properties of samples against an artificial infection background of two major bacterial diseases of soybean, it was found that treatment with Cu^2+^ChiNPs solution of seeds and leaves that had been previously infected by bacterial diseases is an effective tool to reduce pathogen damage in soybean.

These results are encouraging because the studied samples could potentially be used as an element of protection of soybean against the diseases of bacterial etiology mentioned in this study. However, potential side effects on non-target organisms should be evaluated and field trials should be conducted before using substances as pesticides to control phytopathogenic bacteria on an industrial scale.

## Figures and Tables

**Table 1:** Concentrations of samples obtained by dilution of the initial (100% solutions) in distilled water.

Samples	Relative Concentrations of Samples, % (*v/v*)	Concentration of Chitosan, mg/mL	Concentration of CuSO_4_, mg/mL
ChiH	100	5	-
	75	3.75	-
	50	2.5	-
	25	1.25	-
	10	0.5	-
	1	0.05	-
Cu^2+^ChiH	100	5	0.83
	75	3.75	0.62
	50	2.5	0.42
	25	1.25	0.21
	10	0.5	0.083
	1	0.05	0.0083
ChiNPs	100	5	-
	75	3.75	-
	50	2.5	-
	25	1.25	-
	10	0.5	-
	1	0.05	-
Cu^2+^ChiNPs	100	5	0.83
	75	3.75	0.62
	50	2.5	0.42
	25	1.25	0.21
	10	0.5	0.083
	1	0.05	0.0083
CuSO_4_	100	-	0.83
	75	-	0.62
	50	-	0.42
	25	-	0.21
	10	-	0.083
	1	-	0.0083

**Table 2:** Characteristics of ChiNPs and Cu^2+^ChiNPs.

Samples	Size, nm	Polydispersity Index	Zeta-Potential, mV
ChiNPs	254 ± 37	0.499	37.8 ± 1.6
Cu^2+^ChiNPs	153 ± 30	0.421	22.7 ± 0.4
ChiNPs cf *	251 ± 32	0.367	48.5 ± 0.6
Cu^2+^ChiNPs cf *	157 ± 42	0.540	27.2 ± 0.6

*: ChiNPs or Cu^2+^ChiNPs were preliminarily centrifuged for 10 min at 1000× *g*, and then supernatants were centrifuged at 14,000× *g* for 20 min to separate NP fraction.

**Table 3:** Inhibitory and bactericidal concentrations of chitosan samples and CuSO_4_ against Psg and Cff strains.

Samples	Minimal Inhibitory (MIC) and Bactericidal (MBC) Concentrations of Samples, µg/mL (Chitosan/Copper)											
	Psg Strains						Cff Strains					
	CFBP 2214	G2	G17	CFBP 2214	G2	G17	CFBP 3418	F-125-1	F-30-1	CFBP 3418	F-125-1	F-30-1
	MIC			MBC			MIC			MBC		
ChiH	156/-	156/-	156/-	625/-	625/-	625/-	78/-	78/-	78/-	312/-	312/-	312/-
Cu^2+^ChiH	78/13	78/13	78/13	78/13	78/13	39/6	19/3	19/3	19/3	312/52	312/52	0.321/52
ChiNPs	39/-	39/-	39/-	156/-	156/-	156/-	39/-	39/-	39/-	156/-	156/-	156/-
Cu^2+^ChiNPs	19/3	19/3	19/3	78/13	78/13	78/13	19/3	19/3	19/3	78/13	78/13	78/13
CuSO_4_	-/6	-/13	-/3	-/13	-/26	-/13	-/13	-/13	-/13	-/52	-/52	-/52

## Data Availability

The data that support the findings of this study are available on request from the corresponding author.

## Supplementary Materials

The following supporting information can be downloaded at: https://www.mdpi.com/article/10.3390/polym15051100/s1, Figure S1: Primary testing of the antibacterial properties of Cu^2+^Chi-NPs against Psg and Cff strains by agar diffusion. We added 100 µL of solution to the wells, and the inhibition zone was measured after 48 h of incubation at 28 °C. A: growth inhibition of Pseudomonas savastanoi pv. glycinea CFBP 2214; B: growth inhibition of Curtobacterium flaccumfaciens pv. flaccumfaciens; Figure S2: Phytotoxicity of 100% solution Cu^2+^ChiH (B) and water treatment (A) 72 h after treatment of soybean leaves. Characteristic leaves from the groups are presented; Figure S3: Psg and Cff symptoms on soybean leaves 12 d after inoculation with an airbrush. (A) Water treatment of infected leaves (positive control; Psg infection); (B) treatment with Cu^2+^ChiNPs (Psg infection); (C) water treatment of infected leaves (positive control; Cff infection); (D) treatment with Cu^2+^ChiNPs (Cff infection). Characteristic leaves from the groups are presented.
