# Supplementary material for: Protective Properties of Copper-Loaded Chitosan Nanoparticles against Soybean Pathogens Pseudomonas savastanoi pv. glycinea and Curtobacterium flaccumfaciens pv. flaccumfaciens
Source: Polymers (Basel). 2023 Feb 22;15(5):1100. doi: 10.3390/polym15051100 (PMC10007554; doi:10.3390/polym15051100)
Supplement: Supplementary file 1 [file polymers-15-01100-s001.zip › polymers-2232471-supplementary.pdf]

Supplementary materials

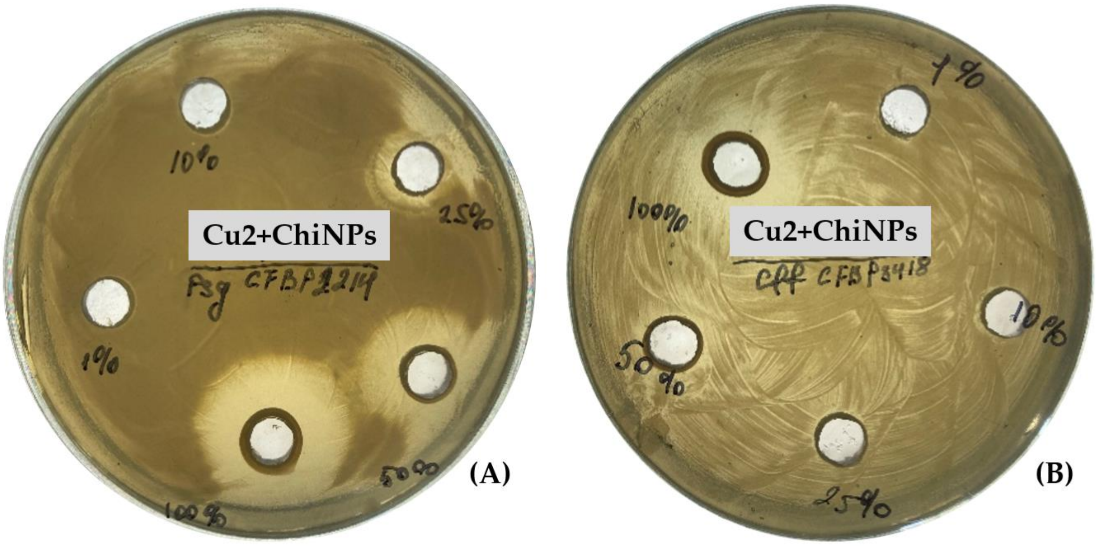

Figure S1. Primary testing of the antibacterial properties of Cu^{2+}Chi-NPs against Psg and Cff strains by agar diffusion. 100 μL of solutions were added to the wells, and the inhibition zone was measured after 48 h of incubation at 28°C. **A**: growth inhibition of *Pseudomonas savastanoi* pv. *glycinea* CFBP 2214; **B**: growth inhibition of *Curtobacterium flaccumfaciens* pv. *flaccumfaciens*.

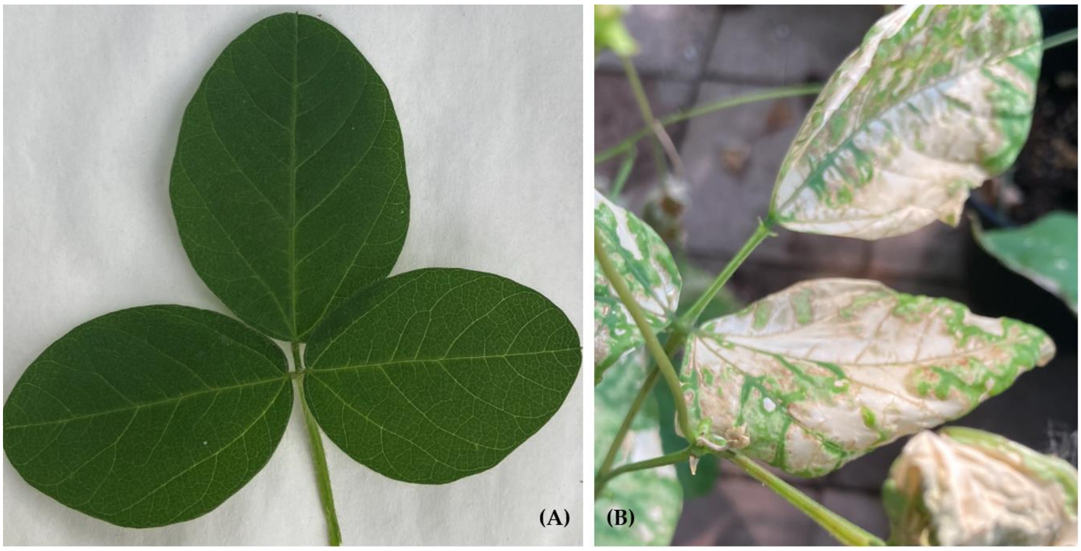

Figure S2. Phytotoxicity of 100% solution Cu^{2+}ChiH (**B**) and water treatment (**A**) 72 h after treatment of soybean leaves. Characteristic leaves from the groups are presented.

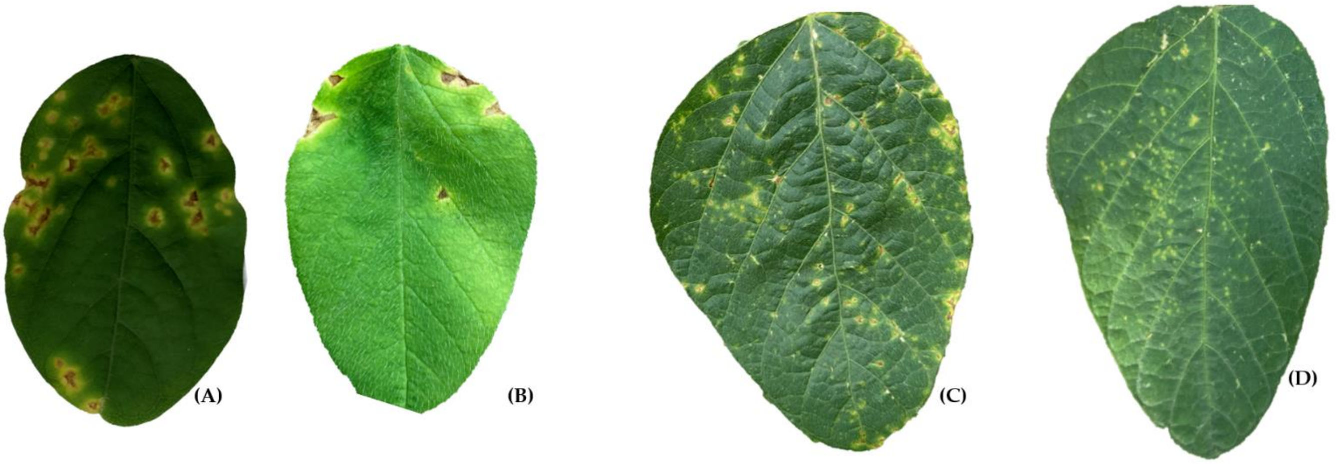

Figure S3. Psg and Cff symptoms on soybean leaves 12 d after inoculation with an airbrush. (**A**) water treatment of infected leaves (positive control; Psg infection); (**B**) treatment with Cu^{2+}ChiNPs (Psg infection); (**C**) water treatment of infected leaves (positive control; Cff infection); (**D**) treatment with Cu^{2+}ChiNPs (Cff infection). The characteristic leaves from the groups are presented.
